# The infectious disease challenges of our time

**DOI:** 10.3389/fpubh.2013.00007

**Published:** 2013-03-22

**Authors:** Jos W. M. van der Meer

**Affiliations:** Department of Internal Medicine, Nijmegen Instutie for Infection Inflammation and Immunity, Radboud University Nijmegen Medical CentreNijmegen, Netherlands

Infectious diseases form one of the greatest global challenges in medicine in our time. Ever since the famous, wrong prediction of the Surgeon General of the United States, William H. Stewart, in 1969: “We can close the books on infectious diseases,” infectious diseases have been on the rise. A large number of new infectious diseases emerged: major threats to the world like AIDS and SARS, but also less threatening infections, like those caused by Campylobacter spp, Borrelia spp, *Bartonella henselae*, *Clostridium difficile*, Hanta viruses. These have led to major changes in clinical practice, microbiology services, public health activities, and biomedical research.

Not only new infectious agents, but also known microorganisms have appeared that acquired new virulence (e.g., the group A streptococcus, and *Staphylococcus aureus*, both causing toxic shock syndrome, the Beijng type of *Mycobacterium tuberculosis*) or new antimicrobial resistance (such as extended-spectrum beta-lactamase and carbapenemase producing Gram-negative bacteria, vancomycin-resistent Gram-positive bacteria and multi-drug resistant Mycobacteria and drug-resistant Plasmodia). The emerging infections reflect the versatile nature of the organisms within the microbial world, and they represent major challenges that will continue to arise for those engaged in research of infectious diseases. This research should not only deal with the mechanisms of microbial plasticity, but also with entirely new ways of intervention, i.e., therapy and prevention. In addition, scientific input is also necessary in epidemiology, including surveillance, early warning and response. With regard to outbreak management, the areas of logistics, decision-making and communication to professionals and to the public have been neglected fields of research. The infectious diseases that occur in the context of poverty form a great challenge. How can we prevent and treat these in an affordable fashion, thereby breaking the cycle of disease, lack of income and increase of poverty, poor living conditions and increased susceptibility to infection?

The new journal, Frontiers in Infectious Diseases, has the ambition to become a major journal to publish the progress of the research mentioned above. In addition, since we know far too little of the pathophysiology of infectious diseases, this is an area of great interest for Frontiers. How do microorganisms adapt to the microenvironment at different body sites? How do microorganisms and the defence of the host modulate in time, in response to each other? Is it possible to obtain real-time information about the state of this war, to guide therapy in clinical medicine? Our *in-vivo* imaging techniques lack discriminative power to obtain this kind of information. Also, our modalities for intervention are limited and rather primitive, mainly derived from traditional *in-vitro* studies. From new insights in pathogenesis of infection, new diagnostic approaches and perhaps new therapies may be derived.

During the past half-century, we disposed of a variety of antimicrobial drugs that really changed the prognosis of infectious diseases in many parts of the world. However, largely by indiscriminate medical and veterinary use of these marvellous drugs, a huge selective pressure has been exerted on the microbial flora that colonizes humans and animals, as well as on the environmental microbes. In the gloomiest scenario, we will be arriving at a situation that is similar to that of the pre-antibiotic era (1). Interestingly, the antimicrobial resistance is not homogeneously spread over the world. Some countries, like the Netherlands and the Scandinavian countries have less severe antimicrobial resistance problems than other countries. Although the reasons for these differences are multi-factorial, a main reason is the prudent use of antibiotics in these countries. Research is needed to better understand the determinants of antibiotic usage in different countries and to come up with methods to change prescribing behavior and patient demand (2).

As resistance to the existing drugs is rapidly increasing, the stagnant development of new antibiotics is of great concern. The tragedy here is that, on the one hand, the classical techniques of antibiotic development that were highly effective in “the golden age of antibiotic discovery” have been abandoned, while, on the other hand the promises of genomic approaches, so far, have not come true (3). In fact, the latter approaches have not led to useful drugs. Do we have to return to the old strategies of antibiotic discovery, using natural resources? There must be sources of antimicrobial (such as plants, marine organisms) that have not been fully explored. But we also need entirely new approaches to combat infection. In this respect, it is tragic that industry lost interest in antibiotics, because of the enormous costs of development and the relatively low chance of return of investment with antibiotics (because they are prescribed mainly in short courses). Here, new strategies, not only in the scientific approach, but also regarding reimbursement incentives are needed.

Another area of tragedy is the immunotherapy of infectious diseases. First of all, all interventions that aim to interfere with the deleterious cytokinemia in sepsis (like antibodies and inhibitors against tumour necrosis factor (TNF), and interleukin-1 receptor antagonist) failed in randomized controlled clinical trials. Why this is, is speculative, but to my mind the following factors—in combination—play a role. First of all sepsis is not one entity. In the past decades, the SIRS (severe inflammatory response syndrome) and sepsis concept as proposed by the late Roger Bone was considered one kind of cytokine disorder (4) in which severity was largely due to the concentrations of deleterious cytokine such as TNF. Non-infectious and infectious causes of SIRS and sepsis were thought to be very similar if not equal regarding the pathophysiological events. This is clearly an oversimplification and the complexity of the cytokine networks was clearly underestimated. For instance, sepsis arising from an infectious focus in the abdomen is different from that in other body sites; recent data obtained with systems biology indicate that different microorganisms differ with regard to the complex cytokine pathways (“signature”) that are being induced (5). Hence certain strategies may work for some microorganisms, while being deleterious for others. On the other side of the coin, human hosts may also differ in the magnitude and the pattern of their responses. This would mean that certain interventions do not work in all patients. Also, the concept of “non-microbial sepsis” has to be revisited with modern techniques, to see whether this is a real entity and to what extent it differs from what infectious disease physicians would coin as sepsis.

Especially in the large clinical trials on sepsis intervention, the heterogeneity of the patients probably has contributed to the poor outcome of these trials. Another serious problem was the poor execution of these mega-trials, with regard to inclusion of patients (6). Without doubt, this has harmed quite a number of new drugs. Another point here is that the mega trials were started before a good assessment of the biological effects of the intervention was done. It has become clear that for instance the administration of anakinra (recombinant interleukin-1 receptor antagonist) was too short, as rebounds were seen after cessation of the drug.

We also embarked on these trials, before it was clear that the proinflammatory state in sepsis was rapidly followed by an anti-inflammatory mode (nowadays called “immunoparalysis”) (7, 8). Most interventions were aimed at the proinflammatory state, and did not take into account this second phase. In fact, currently the attention seems to be directed to the second phase only. It is clear that we need better descriptions of the mediators and of the sequence of events in the various life-threatening infections. Thus, interventional RCTs (randomized controlled clinical trials) remain a challenge in this field, but are feasible in well-defined patient groups; the clarithromycin RCT in septic patients with ventilator-associated pneumonia is a good example (9). Other interventions in carefully defined sepsis patients that are amenable for investigation are interferon-g, and granulocyte macrophage colony stimulating factor treatment.

This brings us to the next challenge. Despite the fact that we have safe recombinant cytokines that are capable to enhance host defence, such as interferon-g, granulocyte colony stimulating factor and granulocyte macrophage colony stimulating factor—with a few exceptions—we do not have solid evidence when to use these drugs in patients. Given the rarity of conditions in which these drugs may be useful (e.g., refractory tuberculosis and other difficult to treat infections), it is difficult, albeit not always impossible, to perform RCTs (10). Apart from RCTs, we will have to consider other methods to assess their therapeutic potential and that of exciting new immunomodulatory drugs that are being developed.

Finally, a major area of success has been the development of vaccines. Although there are a series of effective and non-toxic vaccines available, there are grand challenges ahead, as we have not been able to produce effective vaccines against a number of severe infections (the most prominent being HIV infection). More knowledge of relevant vaccine epitopes, of proper adjuvants and of the protective immune response to candidate vaccines is urgently.

In sum, we should be eager to read the books on infectious diseases instead of closing them; they are full of challenging unread pages, there to be explored… By publishing exciting articles, Frontiers of Infectious Diseases is ready to contribute to this exploration!
